# Mirror Within: Exploring the Impact of Physical Activity on Body Image and Anxiety in Youth

**DOI:** 10.3390/jcm14238484

**Published:** 2025-11-29

**Authors:** Kanupriya Rawat, Aleksandra Błachnio, Hanna Liberska

**Affiliations:** 1 Department of Psychology, Kazimierz Wielki University, 85-064 Bydgoszcz, Poland; alblach@ukw.edu.pl (A.B.); hanna.liberska@op.pl (H.L.); 2 Department of Psychology, University of Zielona Góra, 65-417 Zielona Góra, Poland

**Keywords:** body appreciation, self-esteem, anxiety, physical self-concept, BMI, athletes, youth, mental health

## Abstract

**Background:** Body image, physical self-concept and anxiety are closely intertwined aspects of psychological well-being among youth. The growing influence of social media and appearance-focused culture has intensified self-evaluation pressures, making it essential to understand whether physical activity fosters protective effects or, conversely, contributes to anxiety. **Methods:** The study examined the relationship between body appreciation, physical self-concept, self-esteem, and anxiety among 246 young adults aged 18–35 years (47.6% athletes, 52.4% non-athletes). Participants completed the Rosenberg Self-Esteem Scale (RSES), Body Appreciation Scale-2 (BAS-2), State-Trait Anxiety Inventory (STAI), and the short form of Physical Self-Description Questionnaire (PSDQ-S). Group differences were analyzed using the Mann–Whitney U and Kruskal–Wallis H tests, and associations were explored with Spearman’s correlations. Moderation analyses (PROCESS Model 1) tested whether physical activity buffered BMI-related effects, and structural equation modeling (SEM) evaluated direct and indirect pathways. **Results:** Athletes reported higher self-esteem and body appreciation and scored higher on all PSDQ-S subscales, alongside lower trait anxiety but higher state anxiety than non-athletes. Higher BMI predicted lower self-esteem, body appreciation, and less favorable self-perceptions. Physical activity moderated the BMI—self-esteem and BMI—body appreciation relationships, buffering negative effects among athletes. SEM showed that physical activity positively influenced physical self-concept and body appreciation, which in turn reduced trait anxiety. Gender differences were minimal. **Conclusions:** Regular sport participation supports psychological resilience by enhancing self-esteem and body appreciation while reducing anxiety. However, the findings also highlight the complexity of body–mind dynamics where individuals with strong body appreciation may still experience transient anxiety in evaluative contexts. Promoting body functionality, self-compassion, and positive physical self-concept in educational and sport settings may help prevent maladaptive behaviors and foster lasting mental well-being among youth.

## 1. Introduction

Physical activity plays a fundamental role in promoting holistic health among youth. It strengthens cardiovascular efficiency, builds musculoskeletal fitness, enhances self-esteem, and contributes to better academic and emotional functioning [1,2]. Yet, this generation of young people is also growing up in a visually saturated world, where social media platforms such as Instagram, TikTok, and Snapchat have transformed the body into a central element of self-expression and social identity. Within this visually oriented culture, body appearance has become a dominant measure of self-worth, leading to increased preoccupation with body image and social comparison [3].

Identity development is intricately tied to the construction of a positive body image, a multidimensional construct encompassing perceptions, emotions, and behaviors related to one’s physical appearance and bodily functioning. However, maintaining body satisfaction has become increasingly difficult in a digital culture dominated by idealized images of thinness, muscularity, and “flawless” aesthetics [4]. According to Zaccagni et al. [5], body image dissatisfaction (BID) is one of the most persistent psychosocial issues among young people and is strongly linked with adverse mental and physical health outcomes, such as low self-esteem, depressive symptoms, obesity, disordered eating, reduced participation in physical activity, and impaired physical fitness.

This body–mind interdependence raises a critical question for both clinicians and educators: can physical activity serve as a protective factor against the negative consequences of body dissatisfaction? Empirical evidence increasingly supports this possibility. Engagement in sports encourages a shift from an appearance-based to a functionality-based evaluation of the body, allowing youth to appreciate its capabilities rather than merely its aesthetics [6]. Furthermore, participation in structured physical activities has been associated with better emotional regulation, fewer symptoms of anxiety and depression, and more adaptive coping strategies [1,7]. Despite the potential benefits of physical activity, the relationship between sport participation and body image is not universally positive. Burgon et al. [8] and Izydorczyk et al. [9] highlight that the effects of sport on body image are mediated by multiple contextual factors like training intensity, body-composition monitoring, uniform exposure, and feedback culture. For example, female weightlifters often face a paradox: their strength and physique contradict mainstream ideals of femininity, which can heighten self-consciousness. Youth participating in team sports, on the other hand, tend to exhibit stronger social belonging and less anxiety [10,11]. These nuances demonstrate that physical activity is not a uniform experience; its outcomes depend on the interaction between the athlete’s psychological profile, sport environment, and social feedback.

Youth are exposed to contradictory cultural messages: on one hand, the glorification of slenderness for girls and muscularity for boys; on the other, movements advocating body positivity and neutrality [4,8,12]. This duality often produces ambivalence and instability in self-perception. Findings across developmental and cross-cultural studies indicate that dissatisfaction arises early. Boys frequently experience pressure to increase muscularity, while girls strive for thinness or aesthetic perfection [4,13]. The Polish study on male weight trainers revealed that 62% desired greater muscularity despite already being trained and fit, reflecting the internalization of hyper-muscular ideals [13]. Similarly, aesthetic-sport athletes such as gymnasts, dancers, and swimmers report heightened anxiety and body dissatisfaction, often due to external scrutiny, body-measurement routines, or coach expectations [14,15]. Social media intensifies these pressures. Constant exposure to digitally altered images reinforces unrealistic appearance standards and social comparison, leading to anxiety, low self-esteem, and obsessive body monitoring. Self-objectification processes mediated by online visibility can produce chronic self-surveillance, emotional exhaustion, and disordered eating behaviors [3].

Although physical activity is a known moderator of anxiety, its psychological effects depend on context and body perception. Positive physical self-concept has consistently been associated with greater participation, endurance, and perceived competence among youth [16,17]. Studies conducted among French, Italian, and Chinese adolescents show that a well-developed physical self-concept acts as a buffer against anxiety and promotes overall well-being [18,19]. Conversely, low body satisfaction combined with high physical activity may lead to compulsive exercise or anxiety about weight, especially when appearance rather than health becomes the focus [14]. Petisco et al. [14] reported that competitive athletes reported higher anxiety and perfectionism scores than non-athletes, particularly in sports emphasizing leanness. Similarly, Li et al. [15] concluded that even athletes satisfied with their physique can develop disordered behaviors due to sociocultural and competitive pressures. These findings underscore that physical activity, while beneficial, is not an automatic remedy for anxiety. It must be contextualized within healthy self-perception frameworks.

Given the intertwined nature of body image and anxiety, understanding how youth experience their bodies through physical activity is essential for designing preventive and therapeutic programs. Clinically, promoting body functionality appreciation, valuing what the body can do rather than how it looks has proven to reduce anxiety and disordered behaviors [6]. Educators and health professionals should therefore focus on creating inclusive sport environments emphasizing competence, health, and enjoyment instead of external appearance or competition.

The current study builds on this foundation by examining the relationship between physical activity, body appreciation, physical self-concept, self-esteem, and anxiety among youth classified as athletes (those engaged in regular, performance-oriented training and competition) and non-athletes (who do not participate in organized or competitive sports). Exploring these dynamics can clarify whether higher engagement in physical activity acts as a protective factor for mental health or, conversely, whether performance demands exacerbate anxiety and dissatisfaction.

The present study advances existing research on body image and anxiety in three principal ways. First, it simultaneously compares athletes and non-athletes across a broad set of variables including body appreciation, multidimensional physical self-concept, self-esteem, and both state and trait anxiety within a unified model, rather than examining each construct in isolation. Second, the study rigorously evaluates the moderating effect of physical activity on the relationship between BMI and self-related outcomes by examining the BMI × activity interaction on body appreciation and self-esteem. This approach transcends the examination of simple main effects associated with BMI or sport participation, enabling a more precise elucidation of the complexities inherent in these relationships. Third, using structural equation modeling, the study integrates physical activity, perceived body fat, body appreciation, and anxiety into a coherent framework. This clarifies how positive body image mediates the link between physical activity and lower trait anxiety. Taken together, these contributions offer a nuanced and precise understanding of the conditions under which physical activity serves as a psychological protective factor in young adults.

The main objective of this study was to examine how physical activity, BMI, and gender are related to body appreciation, physical self-concept, self-esteem, and anxiety among young adults. Specifically, four objectives were pursued:

1. To compare psychological outcomes (body appreciation, physical self-concept, self-esteem, state and trait anxiety) across BMI categories.
2. To examine differences in these outcomes between athletes and non-athletes.
3. To assess whether physical activity status moderates the association between BMI and self-related outcomes (body appreciation and self-esteem).
4. To test a structural model wherein physical activity and perceived body fat predict body appreciation, which subsequently relates to state and trait anxiety.

## 2. Materials and Methods

### 2.1. Sample

A total of 246 participants aged 18–35 years took part in the study. The athlete group (*n* = 117; 47.6%) consisted of athletes formally affiliated with sports clubs, while the non-athlete group (*n* = 129; 52.4%) comprised healthy individuals who were not engaged in organized training. Gender distribution was 58.5% female (*n* = 144) and 41.5% male (*n* = 102). According to BMI classification, 73.6% of participants were of normal weight, 17.5% overweight, 6.1% underweight, and 2.8% obese.

The inclusion criteria required that all participants (a) were within the specified age range, (b) were free from any serious illness or condition limiting daily physical activity. Participants were classified based on established definitions distinguishing athletes from non-athletes (exercisers). According to the European Society of Cardiology [20] and later updates [21,22,23], athletes are individuals who regularly train to improve performance, participate in official competitions, and are registered in sports clubs or federations. In contrast, non-athletes (exercisers) engage in physical activity primarily to maintain health, fitness, or physique, without a focus on competitive performance. Specifically, a non-athlete (exerciser) engages in more than 2.5 h per week of physical activity with the primary aim of maintaining health and fitness status [23,24]. All participants were informed about the aims and procedures of the research, the voluntary nature of their participation, and their right to withdraw at any time without consequences. Written informed consent was obtained from every participant. The research was conducted in accordance with the ethical principles of the Declaration of Helsinki. Ethical approval was granted by the University Ethics Committee (Kazimierz Wielki University, Bydgoszcz, Poland) prior to data collection. Data were treated confidentially and analyzed anonymously, ensuring participant privacy and integrity throughout the study.

### 2.2. Measures

Participants self-reported age, gender, height, weight, education, domicile, and sport involvement, frequency of physical activity, hours of physical activity per session approx, quality of physical activity (amateur, national, international or recreational). BMI (kg/m^2^) was calculated and classified into four categories (underweight, normal, overweight, obesity).

The following questionnaires were administered.

#### 2.2.1. Self-Esteem

It was measured using the Rosenberg Self-Esteem Scale (RSES) by M. Rosenberg, in the Polish adaptation by Łaguna et al. [25]. The RSES is a well-validated tool designed to assess global self-esteem understood as an individual’s overall attitude toward the self. It consists of ten statements rated on a 4-point Likert scale ranging from 1 (strongly agree) to 4 (strongly disagree). The total score ranges from 10 to 40 points, where higher scores indicate more positive self-regard. Following standard interpretation guidelines, scores between 10 and 27 reflect low self-esteem, 28–32 medium self-esteem, and 33–40 high self-esteem.

#### 2.2.2. Body Appreciation

It was evaluated using the Body Appreciation Scale-2 (BAS-2) in its Polish adaptation by Razmus and Razmus [26]. The BAS-2 contains ten items that capture self-acceptance, respect, and appreciation of the body’s functionality and appearance (e.g., “I feel good about my body”). Responses are given on a 5-point Likert scale ranging from 1 (never) to 5 (always). Total scores range from 10 to 50, with higher scores denoting greater body appreciation. In the present study, the BAS-2 demonstrated excellent internal consistency reliability (Cronbach’s α = 0.96).

#### 2.2.3. Anxiety

It was assessed using the State-Trait Anxiety Inventory (STAI; Forms X-1 and X-2) in the Polish adaptation by Wrześniewski et al. [27]. The STAI is one of the most widely used self-report tools for evaluating anxiety in both clinical and research contexts. It consists of 40 items, divided equally between two subscales: State Anxiety (STAI-X1), which measures transient anxiety or tension experienced “at this moment,” and Trait Anxiety (STAI-X2), which measures general and stable anxiety disposition. Each item is rated on a 4-point Likert scale from 1 (not at all) to 4 (very much). Possible scores for each subscale range from 20 to 80, with higher values indicating greater anxiety. Sample items include statements such as “I am tense,” “I am worried,” “I feel calm,” and “I feel secure.”

#### 2.2.4. Physical Self-Concept

It was measured using the Short Form of the Physical Self-Description Questionnaire (PSDQ-S) developed by Marsh et al. [28]. This version includes 40 items assessing 11 dimensions of perceived physical self: Health, Coordination, Activity, Body Fat, Appearance, Sport Competence, Strength, Flexibility, Endurance, Global Physical Self-Concept, and Global Self-Esteem. Each item is rated on a 6-point Likert scale from 1 (false) to 6 (true). Higher scores on individual subscales represent more favorable self-perceptions in the corresponding domain. The short form maintains high internal consistency (α = 0.77–0.94 across subscales) and excellent test–retest reliability (r = 0.57–0.90 over one year).

### 2.3. Statistical Analysis

**Figure 1:**
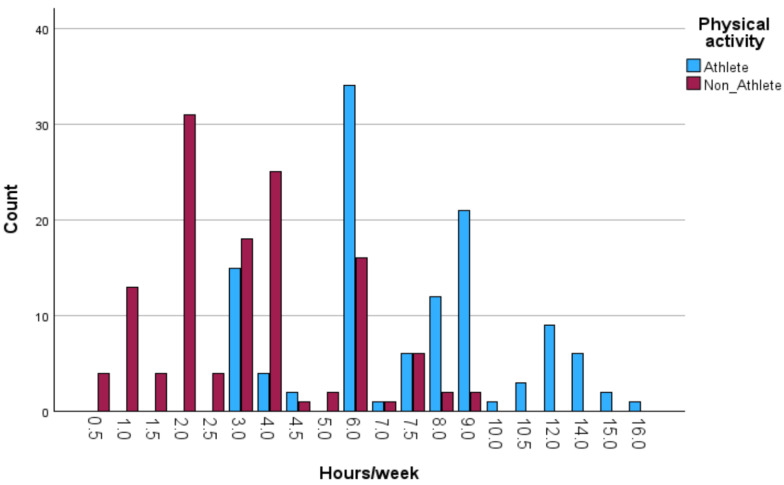
Distribution of hours of physical activity per week among athletes and non-athletes.

**Figure 2:**
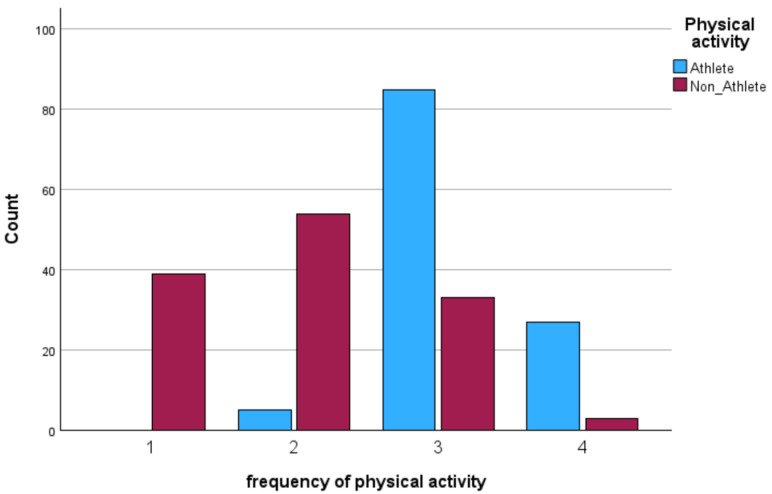
The frequency of physical activity per week (1 = Several days a month, 2 = Once a week, 3 = Several days a week, 4 = Everyday).

**Figure 3:**
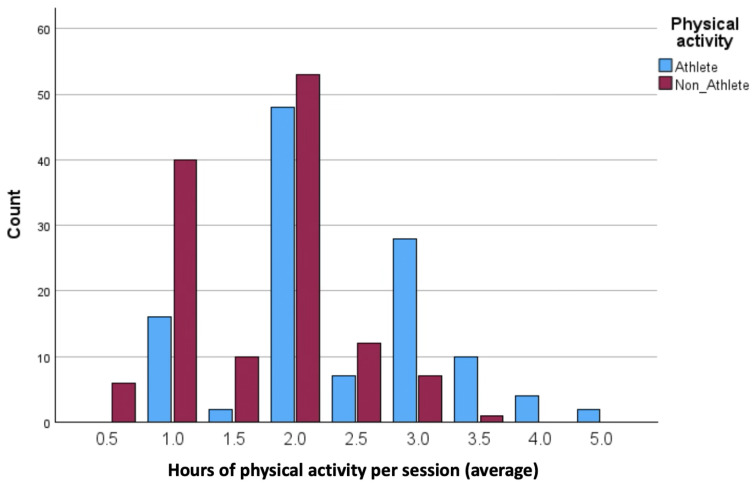
Average duration of physical activity per session.

**Figure 4:**
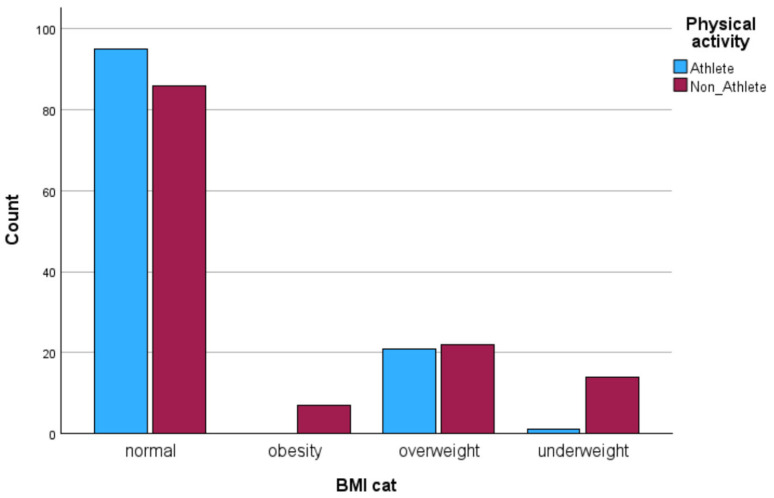
BMI classification of athletes and non-athletes.

IBM SPSS Statistics for Windows version 29.0 and AMOS version 30 were used for the data analysis process. Preliminary screening included checks for missing values, outliers, and the distribution of variables. As the Kolmogorov–Smirnov and Shapiro–Wilk tests indicated that several variables deviated from normality, non-parametric tests were selected for all inferential analyses. Descriptive statistics (mean and standard deviation) were calculated for all variables and presented separately by gender, BMI category, and physical activity group (athletes vs. non-athletes). Visual analyses of physical activity patterns (weekly training volume, session frequency, and duration) were illustrated in Figure 1, Figure 2, Figure 3 and Figure 4.

Group comparisons were performed using three types of non-parametric tests. First, the Kruskal–Wallis H test was used to determine differences in self-esteem, body appreciation, anxiety (state and trait), and the physical self-concept subscales across BMI categories (underweight, normal weight, overweight, and obesity). When significant effects were detected, Bonferroni-adjusted pairwise comparisons were conducted to identify which groups differed significantly. Then the Mann–Whitney U test was applied to examine differences in all psychological scales between physically active (athletes) and non-active (non-athlete) participants, and separately between genders (male and female).

**Figure 5:**
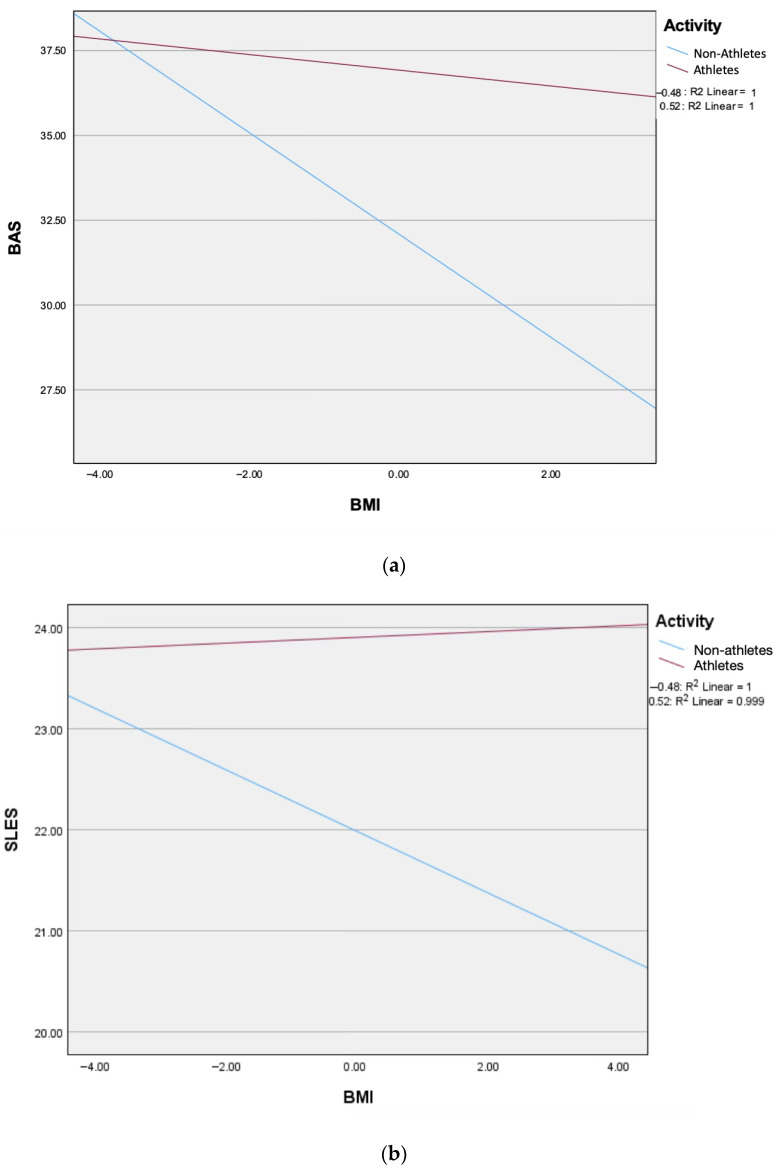
(**a**) Activity x BMI on body appreciation. (**b**) ActivityxBMI on self-esteem.

Moderation analyses were conducted using PROCESS v5.0 (Model 1) to test whether physical activity status moderated the relationship between BMI and (a) self-esteem and (b) body appreciation. Simple slopes and interaction effects were computed and visualized in Figure 5a,b.

Also, a structural equation model (SEM) was estimated in AMOS to examine the direct and indirect effects of physical activity, body-fat perception, and body appreciation on anxiety outcomes. Model fit was evaluated using χ^2^/df, GFI, CFI, NFI, TLI, and RMSEA. Indirect effects were tested using bias-corrected bootstrapping with 5000 samples.

Finally, Spearman’s rank-order correlation coefficient (ρ) was calculated between all primary scales (Rosenberg Self-Esteem, BAS-2, STAI-X1, STAI-X2, and each PSDQ-S subscale) and BMI.

## 3. Results

### 3.1. Characteristics of the Sample

The descriptive statistics results show that the mean age of the participants is 22.78 ± 4.23. The mean value for BMI in underweight is 17.72 ± 0.43, normal is 18.51 ± 1.96, overweight is 26.9 ± 1.52, and obese is 31.96 ± 1.52. The descriptive means and standard deviations for scales are shown in Table 1 for different categories.

Descriptive analyses were conducted to compare the physical activity patterns of athletes and non-athletes. As shown in Figure 1, Figure 2, Figure 3 and Figure 4, athletes demonstrated substantially higher engagement in structured physical activity. Specifically, athletes reported greater weekly training volumes (6–10 h/week), higher frequency of sessions (three to four times per week), and longer session durations (2–3 h). In contrast, non-athletes showed lower overall activity levels, exercising one to two times per week for 1–2 h per session. Regarding BMI classification, most participants in both groups were within the normal-weight range, although underweight and obesity were more frequent among non-athletes.

### 3.2. Differences Across BMI Categories

The Kruskal–Wallis H test revealed several significant differences in psychological and physical self-concept variables across BMI groups (see Table 2).

Specifically, significant group effects were found for self-esteem (H(3) = 11.22, *p* = 0.011) and body appreciation (H(3) = 32.21, *p* < 0.001), indicating that BMI significantly influenced both constructs. Post hoc comparisons (Bonferroni-adjusted) showed that participants with obesity reported significantly lower self-esteem and body appreciation than those with normal weight (*p* < 0.05).

Among the PSDQ-S subscales, significant BMI-related effects emerged for Health (HE; *p* < 0.001), Coordination (CO; *p* = 0.005), Activity (AC; *p* = 0.008), Body Fat (BF; *p* = 0.002), Sport Competence (SP; *p* = 0.006), Global Physical Self-Concept (GP; *p* < 0.001), Appearance (AP; *p* < 0.001), Endurance (EN; *p* = 0.001), and Global Self-Esteem (ES; *p* < 0.001). Participants with normal BMI consistently achieved higher mean ranks in these subscales compared with overweight and obese individuals. No significant BMI differences were observed for Strength (ST; *p* = 0.071) or Flexibility (FL; *p* = 0.361), and state (STAI-X1; *p* = 0.732) and trait anxiety (STAI-X2; *p* = 0.446) did not vary significantly across BMI categories.

### 3.3. Differences by Physical Activity Status

The Mann–Whitney U tests indicated systematic and robust differences between athletes and non-athletes across nearly all variables (see Table 2). Athletes reported significantly higher self-esteem (U = 9543.00, Z = 3.61, *p* < 0.001) and body appreciation (U = 9757.50, Z = 3.97, *p* < 0.001). In terms of anxiety, athletes showed slightly lower trait anxiety (U = 6430.00, Z = –2.01, *p* = 0.045) but notably higher state anxiety (U = 9546.50, Z = 3.60, *p* < 0.001). This pattern suggests that while regular sport participation is associated with greater overall psychological well-being and body-related positivity, competitive environments may temporarily elevate situational anxiety levels.

All PSDQ-S dimensions were significantly higher among athletes, Health (HE; *p* = 0.044), Coordination (CO; *p* = 0.038), Activity (AC; *p* < 0.001), Body Fat (BF; *p* = 0.008), Sport Competence (SP; *p* < 0.001), Global Physical Self-Concept (GP; *p* < 0.001), Appearance (AP; *p* < 0.001), Strength (ST; *p* = 0.004), Flexibility (FL; *p* = 0.037), Endurance (EN; *p* < 0.001), and Global Self-Esteem (ES; *p* = 0.001). These results clearly demonstrate that participation in regular sport activity is associated with more positive self-evaluations, stronger body appreciation, and reduced anxiety.

### 3.4. Moderation Analysis

Moderation analyses were conducted using PROCESS v5.0 (Model 1). As shown in Table 3, activity status significantly moderated the relationships between BMI and both body appreciation (BAS) and self-esteem (SLES).

For self-esteem, activity and BMI were both significant predictors, and the Activity × BMI interaction was significant. This indicates that the effect of activity on self-esteem varies depending on BMI level. As illustrated in Figure 5a, non-athletes showed a clear decrease in self-esteem as BMI increased, whereas athletes showed a slight positive trend. Thus, higher activity levels protected against BMI-related reductions in self-esteem.

For body appreciation, both BMI and activity significantly predicted BAS, and the BMI × activity interaction was significant. As shown in Figure 5b, BMI strongly predicted lower body appreciation among non-athletes, while this effect was substantially weaker and nonsignificant among athletes. This demonstrates that physical activity buffers the negative impact of higher BMI on body appreciation.

Overall, both moderation models revealed significant interaction effects, indicating that being physically active mitigates the negative association between higher BMI and both psychological outcomes.

### 3.5. Structural Equation Model

As shown in Table 4, physical activity (Athletes and Non-Athletes) had significant positive effects on nearly all dimensions of physical self-concept. The strongest relations were observed for appearance, activity, and sport competence, followed by moderate effects on global physical self-concept, endurance, strength, body-fat perception, and global self-esteem. Flexibility also showed a small but significant positive association with activity. Coordination was the only physical self-concept dimension not significantly predicted by activity.

Activity also showed a significant positive effect on body appreciation, whereas perceived body-fat level had only a small, non-significant direct relationship with it. Body appreciation negatively predicted trait anxiety and had a small positive association with state anxiety.

**Figure 6:**
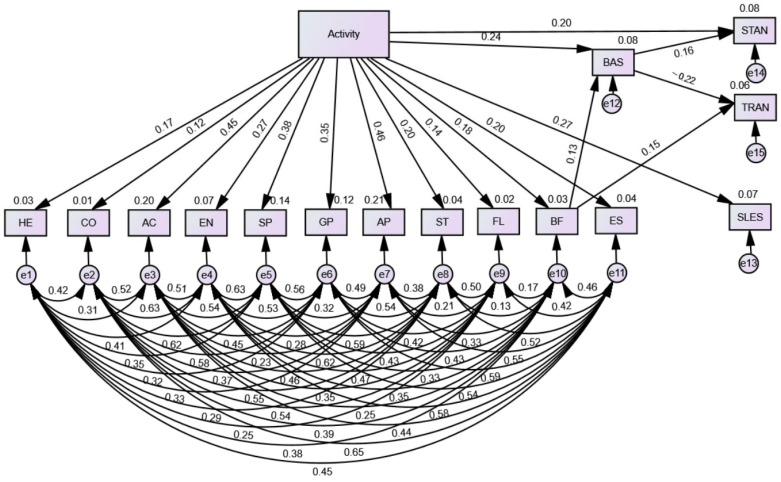
Standardized coefficients of SEM.

Indirect-effect analyses indicated that body appreciation mediated the effects of both physical activity and body-fat perception on trait anxiety. These indirect effects were significant even though the total effects on trait anxiety were not, indicating full mediation through body appreciation. The overall structural model demonstrated acceptable fit (χ^2^ = 82.49, df = 47, (χ^2^/df)  =  1.75, GFI  =  0.959, NFI  =  0.951, CFI  =  0.977, TLI  =  0.942, RMSEA  =  0.056), surpassing the recommended values by Hair, Black [29] (χ^2^/df  <  3, GFI  >  0.9, NFI  >  0.9, CFI  >  0.9, TLI  >  0.9, RMSEA < 0.08). This indicates a favorable fit of the model to the data. Figure 6 illustrates the standardized coefficients for the variables in the SEM.

### 3.6. Gender Differences

Gender-based comparisons using the Mann–Whitney U test revealed few statistically significant differences. Female and male participants did not differ significantly in self-esteem (*p* = 0.238) or body appreciation (*p* = 0.114). However, males reported significantly higher state anxiety (U = 8479.50, Z = 2.07, *p* = 0.038, r = 0.13), whereas trait anxiety did not differ by gender (*p* = 0.303). For physical self-concept, the only gender-related effect emerged for the Activity (AC) subscale, where males scored higher (U = 8779.00, Z = 2.62, *p* = 0.009, r = 0.17), suggesting that male participants perceived themselves as more physically active. No significant gender differences were found for the remaining dimensions of physical self-concept.

### 3.7. Correlation Analysis

Spearman’s rank-order correlations showed meaningful associations among the psychological constructs and BMI. Body appreciation correlated negatively with trait anxiety (ρ = −0.182, *p* = 0.004), indicating that greater body appreciation is linked with lower general anxiety. State anxiety correlated positively but weakly with body appreciation (ρ = 0.162, *p* = 0.011) (see Table 5).

BMI correlated negatively with several variables: body appreciation (ρ = −0.320, *p* < 0.001), health (ρ = −0.238, *p* < 0.001), body fat (ρ = −0.183, *p* = 0.004), sport competence (ρ = −0.172, *p* = 0.007), appearance (ρ = −0.229, *p* < 0.001), global physical self-concept (ρ = −0.206, *p* = 0.001), and global self-esteem (ρ = −0.137, *p* = 0.032). These findings indicate that higher BMI was associated with less favorable body evaluations and lower self-esteem. Within the PSDQ-S subscales, strong positive inter-correlations were observed confirming internal coherence of the physical self-concept construct.

## 4. Discussion

This study examined the relationships among body appreciation, physical self-concept, self-esteem, and anxiety in young adults varying by body mass index, gender, and physical activity status. The results underscore the significant influence of both BMI and sport participation on self-perceptions and emotional well-being, whereas gender differences appeared to be comparatively limited.

Consistent with prior research, higher BMI was associated with less favorable self-evaluations, including reduced self-esteem, lower body appreciation, and poorer perceptions of health, appearance, and athletic competence. These results parallel the findings of Guszkowska and Maziarczyk [13], who reported that body dissatisfaction tends to increase with higher body mass and greater divergence from perceived ideal body shapes. Similarly, studies on Polish adolescents and athletes indicate that excessive weight is often internalized as a psychological burden, affecting confidence, self-presentation, and overall life satisfaction [9]. The observed negative associations between BMI and several dimensions of the PSDQ-S, particularly appearance, sport competence, and global self-concept highlight that physical self-evaluations are influenced not only by functional ability but also by culturally shaped ideals of attractiveness and performance.

A second key finding of this study was the consistent difference between athletes and non-athletes. Athletes reported significantly higher levels of self-esteem, body appreciation, and global physical self-concept consistent with findings by Zartaloudi et al. [30], alongside lower trait anxiety. However, they also exhibited significantly elevated state anxiety, likely reflecting the situational pressures and emotional arousal inherent in training and competition contexts. Research indicates that approximately 70% of athletes experience issues such as sleep disturbances, diminished appetite, and concentration difficulties during these periods [31], which can momentarily hinder performance and compromise physical and psychological health [32]. This pattern underscores the dual nature of sport participation: while it supports long-term psychological well-being and positive self-perception, it may also expose athletes to acute stress responses driven by competition demands. Nevertheless, the overall findings support the view that physical activity is a protective factor for mental health, fostering healthier body attitudes and emotional resilience. Earlier studies similarly document that adolescents regularly engaged in organized sports exhibit greater body satisfaction, enhanced self-confidence, and reduced anxiety and depressive symptoms compared to non-active peers [1,2]. Sport involvement likely fosters feelings of competence, mastery, and social connectedness- core components of positive self-perception according to Self-Determination Theory. Moreover, the robust positive effects observed across all PSDQ-S subscales among athletes confirm that sport experience contributes to a multifaceted and positive understanding of one’s body, encompassing both appearance and functionality.

Through moderation analysis, it is revealed that physical activity substantially changed the way BMI related to body appreciation and self-esteem. Among non-athletes, increases in BMI were strongly associated with reduced body appreciation and declining self-esteem, patterns commonly observed in inactive populations. For athletes, however, these negative associations were either markedly weaker or entirely absent. In the case of body appreciation, higher BMI did not meaningfully reduce positive body attitudes among physically active participants. This suggests that regular physical activity buffers young adults from the psychological consequences typically linked with higher body mass, possibly because sport fosters a functional, competence-based understanding of the body, or it could be that negative mental health outcomes among young adults often derive from distorted body understanding rather than body size itself [33]. Also, the slight upward trend in athletes’ self-esteem at higher BMI further supports the idea that sport involvement promotes body-related self-worth that is less dependent on weight and appearance. Taken together, these moderation effects show that physical activity does not merely improve psychological outcomes directly; it also protects individuals from BMI-related vulnerability [34].

Gender-related differences observed in this study were modest. No significant differences emerged between males and females in self-esteem or body appreciation; however, males exhibited slightly higher levels of state anxiety. This finding diverges from much of the existing literature, which often reports either no gender differences or elevated state anxiety among females [35,36], commonly attributed to greater emotional sensitivity and socialization patterns that facilitate the expression of stress and worry. In competitive sport contexts, however, males may experience comparable or heightened state anxiety due to intensified performance expectations, fear of failure, and social pressure to conform to ideals of success and toughness. These factors are particularly salient among young athletes, who must manage not only athletic demands but also concurrent academic, social, and identity challenges typical of adolescence and emerging adulthood [37]. Elevated competitive anxiety in such contexts often stems from rigorous performance pressures, excessive self-imposed expectations, and an outcome-oriented focus rather than intrinsic motivation or enjoyment [38]. Thus, the modest gender difference in state anxiety observed here likely reflects contextual rather than dispositional influences, highlighting the role of situational pressures in shaping gender-specific anxiety experiences among athletes. Males also rated themselves higher on perceived physical activity levels, consistent with traditional gender norms emphasizing physical competence and vigor; however, these differences were minimal. Overall, the limited gender effects observed suggest that body image concerns and self-evaluations among contemporary youth are becoming increasingly gender-neutral, aligning with recent findings that social media exposure has intensified appearance-related pressures for both sexes [4,8].

A particularly noteworthy finding pertained to the relationship between body appreciation and anxiety. Body appreciation was negatively correlated with trait anxiety, indicating that individuals with greater self-acceptance tend to experience lower chronic anxiety. In contrast, it exhibited a weak positive correlation with state anxiety. This apparent paradox underscores the complex and multifaceted nature of body image functioning. Specifically, trait anxiety reflects a stable predisposition toward worry and emotional tension, whereas state anxiety denotes transient, situation-dependent emotional arousal. Importantly, the SEM results indicated that body appreciation mediated the effects of both physical activity and perceived body fat on trait anxiety, suggesting that a positive body image serves as a pathway through which physical activity enhances emotional well-being. Furthermore, the SEM analysis confirmed that physical activity exerts widespread positive effects on physical self-concept, with particularly strong associations observed for appearance, activity, and sport competence domains. These findings underscore the multidimensional structure of physical self-concept and highlight that both appearance-related and functional self-perceptions contribute meaningfully to global self-worth. Thus, young people with high body appreciation appear to be protected from long-term anxiety tendencies but may still experience transient anxiety in self-referential or evaluative contexts. As suggested by Souilliard et al. [6] and Izydorczyk et al. [9], positive body image does not imply emotional detachment; rather, it enhances resilience and self-regulation in the face of situational stress. The weak positive link between body appreciation and state anxiety may therefore represent healthy emotional engagement or heightened situational awareness rather than maladaptive distress. However, over time, this covariation may pose a potential risk factor for the development of maladaptive behaviors among some young individuals. Previous research indicates that an excessive focus on the body may contribute to less adaptive developmental patterns, including compulsive exercise, disordered eating, or bigorexia [39]. This aligns with our findings, which revealed a significant correlation between state anxiety and body appreciation. Future research should prioritize empirically determining the proportion of young people who maintain a healthy relationship with their body versus those who develop maladaptive behaviors. Addressing this question necessitates longitudinal study designs. Only through such prospective investigations can the temporal dynamics and trajectories of body image and related behaviors be accurately assessed, thereby informing effective prevention and intervention strategies.

The observed inter-correlations among the PSDQ-S subscales further confirm the multidimensionality and internal coherence of physical self-concept. Strong associations between sport competence, appearance, global physical self-concept, and self-esteem are consistent with prior psychometric and empirical studies [16,28], which affirm that these components collectively constitute an integrated perception of physical self-worth. These findings strengthen the argument that both functional (e.g., coordination, endurance) and appearance-related (e.g., body fat, attractiveness) aspects of self-perception significantly contribute to overall body image and emotional adjustment, reinforcing the comprehensive and hierarchical structure of physical self-concept as reflected in validated measurement instruments such as the PSDQ-S.

Taken together, the results provide evidence that regular physical activity supports psychological resilience by enhancing body appreciation and physical self-concept, thereby buffering against anxiety. Teaching younger generations to consciously appreciate their bodies, not only in aesthetic terms but more importantly in terms of physical functionality, can liberate them from the pressure of weight control. It is not the number of kilograms, but physical fitness and self-compassion that play the key role in fostering a healthy and positive attitude toward one’s body. This perspective aligns with a growing body of research emphasizing the importance of body functionality—appreciating what the body can do over its appearance—as a key determinant of psychological well-being and body satisfaction [6,15]. Such an approach encourages resilience against societal appearance ideals and promotes a more holistic, compassionate relationship with one’s body.

Despite its strengths, this study has several limitations. The cross-sectional design precludes causal interpretation of relationships among variables. All measures were self-reported, which may introduce social desirability or recall bias. SES, parental education, and detailed social-media metrics were not collected and should be included in future research to test the robustness of our findings. Additionally, while the age range (18–35 years) captured both emerging and young adults, the sample was predominantly university-affiliated, potentially limiting generalizability. Future studies should consider employing more precise methods such as bioimpedance analysis (BIA) or bioelectrical impedance vector analysis (BIVA), which provide greater accuracy in assessing body composition and cellular health [24]. Longitudinal or experimental designs including broader age and cultural groups are also recommended to clarify causal mechanisms linking physical activity, body appreciation, and anxiety.

The findings underscore the need to integrate body functionality and appreciation training into sport and educational settings. Due to the lack of definitive evidence establishing whether physical activity precedes changes in physical self-image or vice versa [40,41], the complex and potentially reciprocal relationship between these variables warrants further investigation, particularly in younger populations. Coaches, educators, and mental health professionals should emphasize competence, effort, and health rather than idealized appearance. Encouraging youth to develop balanced attitudes toward their bodies, acknowledging both capability and self-acceptance can reduce vulnerability to anxiety and body dissatisfaction. Moreover, the study supports the inclusion of positive body image interventions as part of preventive programs in schools and sports organizations, helping young people sustain psychological well-being in visually and performance-driven environments.

## 5. Conclusions

The present study provides valuable insights into the interconnections between body appreciation, physical self-concept, self-esteem, and anxiety among young adults with varying BMI, gender, and physical activity levels. A significant majority of participants displayed below-average self-esteem scores, indicating a concerning trend that warrants further examination. This pattern raises critical questions for psychological research regarding whether the decline in self-esteem observed among youth represents a novel and enduring phenomenon within image-conscious generations. Is this the price younger generations pay for constant exposure to visual content where unrealistic beauty standards are prevalent, especially on online platforms [42], resulting in unfavorable social comparisons [43]? Addressing this issue is essential to understanding the underlying factors and developing effective interventions to support adolescent mental health and well-being. The findings underscore the critical importance of physical activity and body image. The results demonstrated that regular participation in sport is strongly associated with higher self-esteem, a more positive physical self-concept, stronger body appreciation, and reduced anxiety. Conversely, higher BMI corresponded with poorer body image and diminished emotional well-being. These results confirm that physical activity acts not only as a behavioral health factor but also as a psychological protective mechanism, promoting positive body-related attitudes and enhancing resilience against anxiety and self-doubt.

A particularly meaningful result was the dual relationship between body appreciation and anxiety: a negative correlation with trait anxiety but a weak positive correlation with state anxiety. This suggests that while individuals with higher body appreciation experience less chronic anxiety but may still show temporary emotional activation in specific evaluative or performance situations. Rather than signaling vulnerability, this reflects a healthy and adaptive emotional response: the ability to engage with one’s experiences without long-term distress.

These findings hold important implications for clinical and educational practice. Promoting body appreciation and positive physical self-concept should become an integral part of youth mental health and sport psychology interventions. Programs that focus on body functionality, self-acceptance, and intrinsic motivation rather than appearance or performance alone can significantly reduce anxiety and improve self-esteem. Coaches, physical education teachers, and mental health professionals should adopt supportive language emphasizing effort, health, and competence over aesthetic ideals. Furthermore, integrating psychoeducation on body image and emotional regulation into sport and school curriculum could help young people internalize balanced and realistic views of their bodies.

In clinical contexts, enhancing body appreciation may complement existing therapeutic approaches for anxiety prevention and stress management. Techniques such as mindfulness, self-compassion training, and positive body-focused cognitive restructuring could be effectively incorporated into counseling or sport-based interventions.

In summary, this study underscores that a positive relationship with one’s body is grounded in appreciation, competence, and acceptance which acts as a cornerstone of psychological well-being. Encouraging youth to value their bodies for what they can do, rather than how they appear, is a vital step toward fostering enduring emotional health, confidence, and life satisfaction.

## Figures and Tables

**Table 1:** Descriptive statistics.

Variables	Gender		BMI				Physical Activity	
	F	M	UN	N	OV	OB	ATH	NATH
SES	22.7 (3.3)	23.3 (3.8)	23.5 (2.4)	23.1 (3.4)	22.8 (3.6)	17.0 (4.8)	23.9 (3.9)	22.0 (2.9)
BAS-2	35.2 (9.2)	33.2 (9.6)	38.7 (9.4)	35.7 (8.8)	29.7 (8.8)	19.0 (2.2)	36.9 (7.1)	32.1 (10.6)
STAI X-1	48.5 (4.3)	49.5 (4.6)	49.0 (3.8)	48.9 (4.4)	49.3 (5.0)	47.0 (4.3)	50.1 (4.3)	47.9 (4.3)
STAI X-2	49.7 (6.6)	48.6 (6.5)	48.6 (4.4)	49.6 (6.8)	48.7 (6.3)	45.9 (6.0)	48.4 (6.8)	50.0 (6.2)
HE	23.5 (6.6)	23.8 (6.0)	23.7 (6.9)	24.8 (5.7)	20.1 (6.5)	13.6 (2.6)	24.8 (5.1)	22.6 (7.1)
CO	21.9 (5.5)	21.9 (5.5)	21.3 (5.8)	22.6 (5.2)	19.9 (5.8)	17.4 (4.5)	22.6 (5.6)	21.3 (5.3)
AC	15.9 (5.3)	17.6 (5.4)	13.6 (5.1)	17.2 (5.1)	15.6 (6.4)	12.9 (3.0)	19.1 (4.0)	14.3 (5.5)
BF	12.4 (4.6)	12.4 (4.5)	13.3 (4.5)	12.9 (4.3)	10.8 (4.9)	7.6 (0.8)	13.3 (4.1)	11.6 (4.8)
SP	11.9 (3.8)	12.7 (3.7)	11.3 (4.3)	12.7 (3.5)	11.1 (4.3)	9.3 (4.1)	13.8 (2.8)	10.9 (4.1)
GP	12.7 (3.5)	12.8 (3.3)	12.0 (4.5)	13.5 (2.8)	10.1 (4.1)	10.9 (2.6)	14.0 (2.6)	11.6 (3.7)
AP	12.4 (3.4)	13.0 (2.9)	12.5 (4.4)	13.3 (2.6)	11.1 (3.7)	6.6 (1.5)	14.2 (2.5)	11.3 (3.1)
ST	11.6 (3.8)	12.2 (3.6)	10.7 (4.2)	12.2 (3.4)	11.3 (4.5)	9.0 (3.0)	12.6 (3.4)	11.1 (3.8)
FL	11.8 (3.9)	11.8 (4.0)	10.8 (4.5)	12.1 (3.9)	11.1 (4.1)	11.0 (2.2)	12.4 (3.9)	11.3 (3.9)
EN	10.8 (4.2)	11.4 (4.4)	8.6 (4.3)	11.7 (4.0)	9.8 (4.7)	7.7 (2.6)	12.3 (4.0)	9.9 (4.3)
ES	21.1 (5.7)	21.3 (6.0)	20.1 (5.5)	22.2 (5.3)	18.2 (6.9)	14.6 (3.2)	22.4 (5.4)	20.0 (6.0)

SES: Self-esteem Scale, BAS: Body Appreciation scale-2, STAI X-1, X2: State and Trait Anxiety Inventory, HE: Health, CO: Coordination, AC: Activity, BF: Body fat, SP: Sport competence, GP: Global physical self-concept, AP: Appearance, ST: Strength, FL: Flexibility, EN: Endurance, ES: Global self-esteem, F: Female, M: Male, BMI: Body mass index, N: Normal, OB: Obesity, OV: Overweight, UN: Underweight, ATH: Athletes, NATH: Non-Athletes.

**Table 2:** Differences in Self-Esteem, Body Appreciation, Anxiety, and Physical Self-Concept Across BMI Categories and Physical Activity Status.

Variable/Scale	BMI				Physical Activity	
	H(3)	*p*	Effect Size	U/Z	*p*	r
SES	11.22	0.011 *	0.033	9543/3.61	<0.001 ***	0.23
BAS-2	32.21	<0.001 ***	0.122	9757/3.97	<0.001 ***	0.25
STAI-X1	1.29	0.732	0	9457/3.6	<0.001 ***	0.23
STAI-X2	2.67	0.446	0.006	6430/2.01	0.045 *	0.13
HE	30.64	<0.001 ***	0.117	8661/2.01	0.044 *	0.13
CO	12.92	0.005 **	0.040	8700/2.07	0.038 *	0.13
AC	11.79	0.008 **	0.035	11,378/6.89	<0.001 ***	0.44
BF	15.34	0.002 **	0.048	9027/2.67	0.008 **	0.17
SP	12.53	0.006 **	0.038	10,730/5.73	<0.001 ***	0.37
GP	31.09	<0.001 ***	0.119	10,647/5.59	<0.001 ***	0.36
AP	30.58	<0.001 ***	0.117	11,745/7.57	<0.001 ***	0.48
ST	7.03	0.071	0.020	9132/2.85	0.004 **	0.18
FL	3.21	0.361	0.009	8708/2.09	0.037 *	0.13
EN	15.43	0.001 **	0.048	9820/4.09	<0.001 ***	0.26
ES	24.87	<0.001 ***	0.079	9345/3.23	0.001 **	0.21

SES: Self-esteem Scale, BAS: Body Appreciation scale-2, STAI X-1, X2: State and Trait Anxiety Inventory, CO: Coordination, AC: Activity, BF: Body fat, SP: Sport competence, GP: Global physical self-concept, AP: Appearance, ST: Strength, FL: Flexibility, EN: Endurance, ES: Global self-esteem. *** *p* < 0.001, ** < 0.01, * *p* < 0.05.

**Table 3:** Moderation Effects of Activity and BMI on Self-Esteem (SLES) and Body Appreciation (BAS).

Outcome Variable	Predictor	ΔR^2^	F	β	*p*	95% CI
Self-esteem	Activity	0.021	5.898 *	1.923	<0.001 ***	[1.086, 2.760]
	BMI	-	-	−0.146	0.031 *	[−0.278, −0.013]
	ActivityxBMI	-	-	0.332	0.016 *	[0.063, 0.601]
Body Appreciation	Activity	0.44	14.41 *	−0.895	<0.001 ***	[−1.218, −0.571]
	BMI	-	-	4.893	<0.001 ***	[2.85, 6.937]
	ActivityxBMI	-	-	0.61	0.0002 ***	[0.61, 1.925]

ΔR^2^ = change in R^2^ due to the interaction term. β = unstandardized regression coefficient from PROCESS. *** *p* < 0.001, * *p* < 0.05.

**Table 4:** Structured Equation Model.

Path	Effect Type	β Coefficient (Standardized)	SE	*p*	95% CI
Activity → HE	Direct	0.173	0.058	0.004 **	[0.056, 0.285]
Activity → CO	Direct	0.122	0.064	0.073 ns	[−0.010, 0.242]
Activity → AC	Direct	0.449	0.049	0.001 ***	[0.348, 0.542]
Activity → EN	Direct	0.273	0.059	0.000 ***	[0.153, 0.383]
Activity → SP	Direct	0.380	0.052	0.000 ***	[0.273, 0.476]
Activity → GP	Direct	0.352	0.056	0.001 ***	[0.235, 0.457]
Activity → AP	Direct	0.463	0.050	0.000 ***	[0.361, 0.556]
Activity → ST	Direct	0.203	0.060	0.001 **	[0.079, 0.320]
Activity → FL	Direct	0.141	0.063	0.027 *	[0.013, 0.261]
Activity → BF	Direct	0.179	0.062	0.004 **	[0.061, 0.299]
Activity → ES	Direct	0.205	0.060	0.002 **	[0.084, 0.318]
Activity → SLES	Direct	0.270	0.050	0.000 ***	[0.165, 0.363]
Activity → BAS	Direct	0.235	0.059	0.000 ***	[0.117, 0.350]
BF → BAS	Direct	0.126	0.065	0.051 ns	[−0.001, 0.251]
BAS → TRAN	Direct	−0.220	0.064	0.001 ***	[−0.347, −0.093]
BF → TRAN	Direct	0.150	0.059	0.013 *	[0.031, 0.260]
BAS → STAN	Direct	0.156	0.064	0.016 *	[0.027, 0.276]
Activity → TRAN	Indirect (via BAS)	−0.030	0.025	0.187 ns	[−0.086, 0.014]
BF → TRAN	Indirect (via BAS)	−0.028	0.016	0.029 *	[−0.069, −0.003]
Activity → STAN	Indirect (via BAS)	0.040	0.019	0.010 *	[0.009, 0.085]
Activity → TRAN	Total	−0.030	0.025	0.187 ns	[−0.086, 0.014]
BF → TRAN	Total	0.122	0.061	0.053 ns	[−0.002, 0.239]
Activity → STAN	Total	0.245	0.057	0.000 ***	[0.129, 0.352]

β = standardized coefficient; SE = standard error; CI = bias-corrected 95% confidence interval (5000 bootstraps). HE: Health, CO: Coordination, AC: Activity, BF: Body fat, SP: Sport competence, GP: Global physical self-concept, AP: Appearance, ST: Strength, FL: Flexibility, EN: Endurance, ES: Global self-esteem, SLES: Self-esteem Scale, BAS: Body Appreciation scale-2, STAN: State Anxiety, TRAN: Trait Anxiety. *** *p* < 0.001, ** < 0.01, * *p* < 0.05, ns- non-significant.

**Table 5:** Correlation.

*n* = 246		1	2	3	4	5	6	7	8	9	10	11	12	13	14	15
1	SES	-														
2	BAS-2	0.11 (0.1)	-													
3	STAI-X1	0.09 (0.15)	0.162 * (0.01)	-												
4	STAI-X2	−0.11 (0.08)	−0.18 ** (0.004)	0.15 * (0.018)	-											
5	HE	0.11 (0.08)	0.18 * (0.003)	0.06 (0.3)	0.015 (0.81)	-										
6	CO	0.06 (0.31)	0.15 * (0.01)	0.06 (0.33)	−0.09 (0.14)	0.42 ** (< 0.001)	-									
7	AC	0.10 (0.1)	0.2 ** (0.001)	0.14 * (0.01)	−0.06 (0.3)	0.32 ** (< 0.001)	0.54 ** (<0.001)	-								
8	BF	0.07 (0.22)	0.18 ** (0.004)	0.12 * (0.047)	0.107 (0.09)	0.37 ** (<0.001)	0.42 ** (<0.001)	0.29 ** (<0.001)	-							
9	SP	0.19 ** (0.003)	0.15 * (0.017)	0.16 * (0.01)	−0.01 (0.88)	0.34 ** (<0.001)	0.64 ** (<0.001)	0.63 ** (<0.001)	0.38 * (<0.001)	-						
10	GP	0.12 * (0.04)	0.22 ** (<0.001)	0.14 * (0.02)	−0.02 (0.72)	0.33 * (<0.001)	0.59 ** (<0.001)	0.54 ** (<0.001)	0.46 ** (<0.001)	0.61 ** (<0.001)	-					
11	AP	0.22 ** (<0.001)	0.23 ** (<0.001)	0.22 ** (<0.001)	−0.04 ** (0.52)	0.32 ** (<0.001)	0.36 ** (<0.001)	0.38 ** (<0.001)	0.35 ** (<0.001)	0.42 ** (<0.001)	0.53 ** (<0.001)	-				
12	ST	0.07 (0.21)	0.04 (0.46)	0.12 * (0.49)	−0.04 (0.53)	0.29 ** (<0.001)	0.57 ** (<0.001)	0.51 ** (<0.001)	0.17 ** (0.007)	0.61 ** (<0.001)	0.55 ** (<0.001)	0.38 ** (<0.001)	-			
13	FL	0.02 (0.65)	0.04 (0.51)	0.06 (0.31)	−0.07 (0.22)	0.26 ** (<0.001)	0.54 ** (<0.001)	0.37 ** (<0.001)	0.19 ** (0.002)	0.44 ** (<0.001)	0.45 ** (<0.001)	0.25 ** (<0.001)	0.5 ** (<0.001)	-		
14	EN	0.06 (0.29)	0.18 ** (0.004)	0.103 (0.106)	−0.106 (0.096)	0.37 ** (<0.001)	0.64 ** (<0.001)	0.56 ** (<0.001)	0.36 ** (<0.001)	0.66 ** (<0.001)	0.55 ** (<0.001)	0.33 ** (<0.001)	0.65 ** (<0.001)	0.48 ** (<0.001)	-	
15	ES	0.07 (0.25)	0.09 (0.15)	0.07 (0.24)	−0.019 (0.77)	0.45 ** (<0.001)	0.66 ** (<0.001)	0.51 ** (<0.001)	0.47 ** (<0.001)	0.56** (<0.001)	0.59 ** (<0.001)	0.52 ** (<0.001)	0.52 ** (<0.001)	0.43 ** (<0.001)	0.43 ** (<0.001)	-
16	BMI	−0.1 (0.09)	−0.32 ** (<0.001)	−0.03 (0.62)	−0.09 (0.12)	−0.23 ** (<0.001)	−0.12 (0.05)	−0.004 (0.94)	−0.18 ** (0.004)	−0.17 ** (0.007)	−0.21 ** (0.001)	−0.22 ** (<0.001)	−0.05 (0.39)	−0.034 (0.59)	−0.06 (0.32)	−0.13 * (0.03)

** Correlation is significant at the 0.01 level (2-tailed). * Correlation is significant at the 0.05 level (2-tailed).

## Data Availability

The original data presented in the study are openly available in Mendeley Data at DOI: 10.17632/xc6p9j7zwz.1.
